# Sharing Mechanical Ventilator: In Vitro Evaluation of Circuit Cross-Flows and Patient Interactions

**DOI:** 10.3390/membranes11070547

**Published:** 2021-07-20

**Authors:** Sebastiano Maria Colombo, Michele Battistin, Eleonora Carlesso, Luigi Vivona, Fabio Carfagna, Carlo Valsecchi, Gaetano Florio, Luca Carenzo, Tommaso Tonetti, Vito Marco Ranieri, Maurizio Cecconi, Antonio Pesenti, Giacomo Grasselli, Alberto Zanella

**Affiliations:** 1 Department of Anaesthesia and Intensive Care Medicine, Fondazione IRCCS Ca’ Granda Ospedale Maggiore Policlinico, 20122 Milan, Italy; sebastiano.colombo@gmail.com (S.M.C.); carlovalsecchi5@gmail.com (C.V.); antonio.pesenti@unimi.it (A.P.); giacomo.grasselli@unimi.it (G.G.); 2 Department of Pathophysiology and Transplantation, University of Milan, 20122 Milan, Italy; eleonora.carlesso@unimi.it (E.C.); luigi.vivona@unimi.it (L.V.); g.floriomed@gmail.com (G.F.); 3 Fondazione IRCCS Ca’ Granda Ospedale Maggiore Policlinico, Center for Preclinical Research, 20122 Milan, Italy; battistin.michele@gmail.com; 4 Department of Anaesthesia and Intensive Care Medicine Rozzano, IRCCS Humanitas Research Hospital, 20089 Milano, Italy; fabio.carfagna@hunimed.eu (F.C.); luca.carenzo@gmail.com (L.C.); maurizio.cecconi@hunimed.eu (M.C.); 5 Dipartimento di Scienze Mediche e Chirurgiche Bologna, Alma Mater Studiorum University of Bologna, 40126 Bologna, Italy; tommaso.tonetti@unibo.it (T.T.); m.ranieri@unibo.it (V.M.R.)

**Keywords:** COVID-19, SARS-CoV2 infection, cross-flow dynamics, shared ventilation, sharing mechanical ventilator, two patients one ventilator

## Abstract

During the COVID-19 pandemic, a shortage of mechanical ventilators was reported and ventilator sharing between patients was proposed as an ultimate solution. Two lung simulators were ventilated by one anesthesia machine connected through two respiratory circuits and T-pieces. Five different combinations of compliances (30–50 mL × cmH_2_O^−1^) and resistances (5–20 cmH_2_O × L^−1^ × s^−1^) were tested. The ventilation setting was: pressure-controlled ventilation, positive end-expiratory pressure 15 cmH_2_O, inspiratory pressure 10 cmH_2_O, respiratory rate 20 bpm. Pressures and flows from all the circuit sections have been recorded and analyzed. Simulated patients with equal compliance and resistance received similar ventilation. Compliance reduction from 50 to 30 mL × cmH_2_O^−1^ decreased the tidal volume (V_T_) by 32% (418 ± 49 vs. 285 ± 17 mL). The resistance increase from 5 to 20 cmH_2_O × L^−1^ × s^−1^ decreased V_T_ by 22% (425 ± 69 vs. 331 ± 51 mL). The maximal alveolar pressure was lower at higher compliance and resistance values and decreased linearly with the time constant (r² = 0.80, *p* < 0.001). The minimum alveolar pressure ranged from 15.5 ± 0.04 to 16.57 ± 0.04 cmH_2_O. Cross-flows between the simulated patients have been recorded in all the tested combinations, during both the inspiratory and expiratory phases. The simultaneous ventilation of two patients with one ventilator may be unable to match individual patient’s needs and has a high risk of cross-interference.

## 1. Introduction

The recent COVID-19 outbreak required a drastic increase in hospital and intensive care unit (ICU) capacity to face the surge of patients requiring respiratory support [1]. In particular, the need for invasive mechanical ventilation was extraordinarily high, leading to a shortage of mechanical ventilators [2]. Manual ventilation with the self-inflating bag is a temporary solution but requires additional personnel and may expose the patients to uncontrolled tidal volume (V_T_) and airway pressures [3,4,5]. The use of one mechanical ventilator for multiple patients was suggested to face emergency contexts [6].

After 20 February 2020, Northern Italy was severely affected by the pandemic, and the number of ICU beds more than doubled in a few weeks [7,8] The number of patients with COVID-19 respiratory failure requiring invasive mechanical ventilation was extraordinarily high and ventilator sharing was hypothesized as a possible temporary intervention [4].

Although sharing a mechanical ventilator could be life-saving [9], several complications have been described [10,11]. Under these circumstances, some societies advised against the use of this technique [1,12]. However, at the time we are writing, Beitler and colleagues have already documented the application of 48 h of ventilator sharing among three patient pairs with COVID-19-associated acute respiratory distress syndrome [9]. Neuromuscular blockade and the careful selection of compatible patients was essential. No adverse events were reported.

Recently, many efforts have been made to characterize ventilation sharing. In addition, ventilator circuit modifications and monitoring have been proposed to provide personalized ventilation and improve the safety profile [13,14,15,16,17]. The ethical aspects related to ventilator sharing are still under discussion [18,19,20,21].

This in vitro evaluation of using one mechanical ventilator for two patients was conceived in such an emergency context [22]. This study aims to increase the knowledge on the interactions and cross-flows between patients, which have been poorly studied so far.

To this end, we evaluated the flows, pressures and volumes in different sections of the ventilator circuits during pressure-controlled ventilation of two patient simulators with different respiratory mechanics. We tested a simple ventilator circuit that can be easily and quickly assembled in any ICU to share a mechanical ventilator.

## 2. Methods

### 2.1. Laboratory Setting

**Figure 1:**
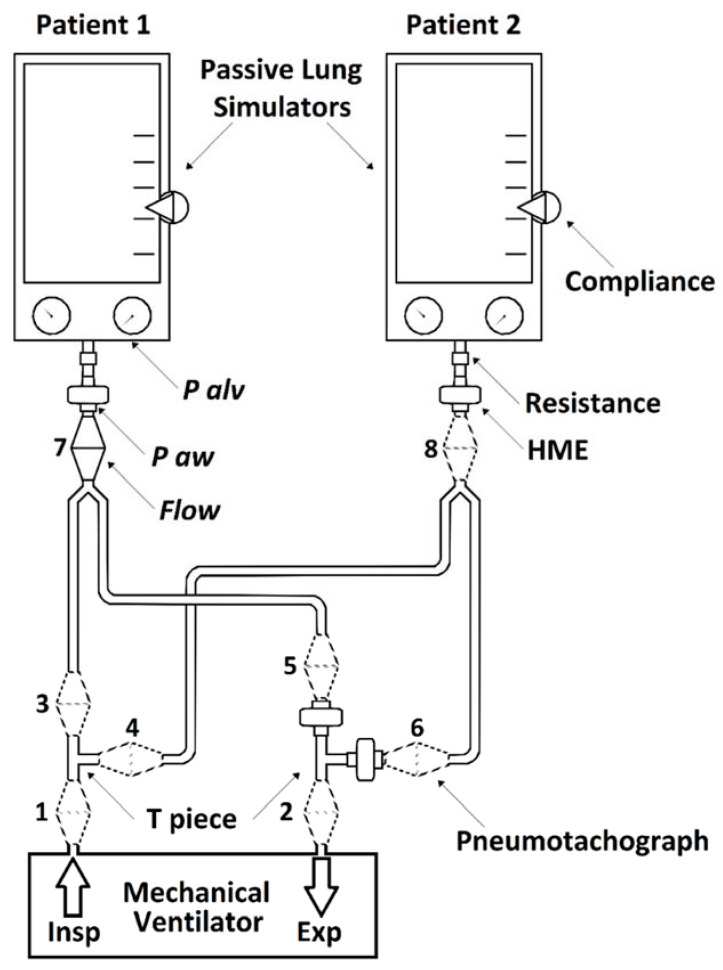
A schematic representation of the circuit tested. P alv = alveolar pressure (cmH_2_O), P aw = airway pressure (cmH_2_O), Flow = flow (L × s^−1^), HME = heat and moisture exchanger, Insp = inspiratory, Exp = expiratory. Numbers indicate flows recorded in each section of the circuit: (**1**) inspiratory branch of ventilator (before T-piece); (**2**) expiratory branch of ventilator (after T-piece); (**3**) inspiratory branch of simulated patient 1 (after T-piece); (**4**) inspiratory branch of simulated patient 2 (after T-piece); (**5**) expiratory branch of simulated patient 1 (before T-piece and HME); (**6**) expiratory branches of simulated patient 2 (before T-piece and HME); (**7**) Y-connector of simulated patient 1 (before HME); (**8**) Y-connector of simulated patient 2 (before HME).

A Mindray WATO EX-65 ventilator (Mindray Medical, Shenzhen, China) was connected to a custom-made ventilator circuit to allow the ventilation of two adult lung simulators (“simulated patient”, Dual Adult Lung Simulator, Michigan Instruments, Grand Rapids, MI, USA). Two adult respiratory circuits for anesthesia (22 mm Smoothbore breathing system, 2 m, Intersurgical, Mirandola, Italy) were connected through 2 T-pieces to the inspiratory and expiratory branches of the ventilator, see Figure 1. HME filters (Inter-Therm Filter/HME, Intersurgical, Mirandola, Italy) were placed at the Y-connectors and expiratory branches of both simulated patients. No one-way valves were used.

One ventilation setting was tested: pressure-controlled ventilation (PCV), positive end-expiratory pressure (PEEP) 15 cmH_2_O, inspiratory pressure of 10 cmH_2_O, respiratory rate (RR) 20 breaths per minute, inspiratory–expiratory ratio 1:2.

Five couples of simulated patients with different combinations of compliance (C: 30 or 50 mL × cmH_2_O^−1^) and airways resistance (R: 5 or 20 cmH_2_O × L^−1^ × s^−1^) were tested, see Table 1. The time constant (τ), i.e., the time necessary to inflate 63.2% of the final volume, was computed as the product of resistance and compliance τ = R × C (s).

For each combination, we measured 4 pressures and 8 flows, see Figure 1. In particular, airway pressures were recorded at the HME at the Y-connectors of each simulated patient, while alveolar pressures of each simulated patient were recorded through the specific port of the lung simulator. Flows were recorded in each section of the custom-made circuit:

1. Inspiratory branch of ventilator (before T-piece);
2. Expiratory branch of ventilator (after T-piece);
3. Inspiratory branch of simulated patient 1 (after T-piece);
4. Inspiratory branch of simulated patient 2 (after T-piece);
5. Expiratory branch of simulated patient 1 (before T-piece and HME);
6. Expiratory branches of simulated patient 2 (before T-piece and HME);
7. Y-connector of simulated patient 1 (before HME);
8. Y-connector of simulated patient 2 (before HME);

All pressures and flows were recorded using 4 pressure sensors (TruWave, Edwards Lifesciences, Irvine, CA, USA) and 1 pneumotachograph (S300 ADInstruments, Bella Vista, Australia) connected to a PowerLab 16/35 (ADInstruments, Bella Vista, Australia).

As we had only 1 pneumotachograph, each setting was repeated 8 times to record for at least 1 min the flows in all the 8 required circuit sections.

### 2.2. Graphical Representation and Statistical Analysis

Volume tracings were computed integrating flow measured by pneumotachograph over time. In order to compare tracings recorded in the different sections of the circuit and between the 2 simulated patients, tracings were normalized with respect to time to compute ensemble averages over multiple breaths recorded and to obtain a single “average” breath for each ventilatory setting, section and simulated patient (the detailed description of the procedure is reported elsewhere [23]). Breaths were resampled by linear interpolation to obtain 500 overlapped samples within the respiratory cycle identified by two consecutive beginnings of the inspiratory cycle. The ensemble averages of flow, volumes, airway and alveolar pressures were computed and expressed as a percentage of the total mean of the respiratory cycle (1 point every 0.2% of the total time). Pressure tracings were analyzed with the pneumotachograph positioned on the inspiratory branch of the circuit to avoid different pressure drops due to the presence of the pneumotachograph in the different sections of the circuit.

In the average breath, we identified: inspiratory and expiratory peak flows at Y-connectors, peak inspiratory flows at the inspiratory branches of the simulated patient and of the ventilator, peak expiratory flows at the expiratory branches of the simulated patient and of the ventilator. Minimum and maximum airway and alveolar pressure were also recorded. Inspiratory volumes at Y-connectors were identified as the maximal volumes at end inspiration. Inspiratory volumes in the other sections of the circuit were identified as the volumes of those sections at the same timepoint. Expiratory volumes were computed as the difference between inspiratory volumes and the volumes at end-expiration for each section. Cross-volumes were computed, during inspiration, as the volume that reached the patient through the expiratory branch, while, during expiration, as the volume expired by the patient through the inspiratory branch. Values were reported as mean ± standard deviation.

T-test was used to compare average values identified on the average tracings between the 2 simulated patients.

## 3. Results

### 3.1. Relationships between Pressures, Volumes and Patients’ Mechanical Characteristics

The average V_T_ recorded at the Y-connectors of the simulated patient, the average minimum and maximum values of alveolar pressures and cross-volumes of the two simulated patients in the five different combinations of compliance/resistance tested in the study are reported in Table 2.

Considering all settings together, we found that V_T_ (at any resistance value) decreased when compliance was reduced from 50 to 30 mL × cmH_2_O^−1^ (418 ± 49 vs. 285 ± 17 mL respectively, a reduction of 32%) and when resistance was increased (at any compliance value) from 5 to 20 cmH_2_O × L^−1^ × s^−1^ (425 ± 69 vs. 331 ± 51 mL respectively, a reduction of 22%).

### 3.2. Setting C 50-50/R 5-5

**Figure 2:**
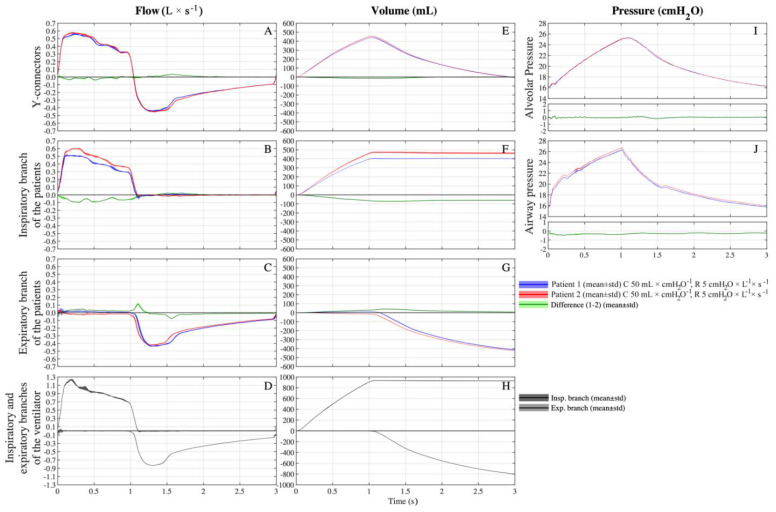
Average (continuous line) ± standard deviation (filled area plot) of multiple breaths of flows, pressures and volumes across the custom-made circuit on setting C 50-50/R 5-5 (C = 50 mL × cmH_2_O^−1^ and R = 5 cmH_2_O × L^−1^ × s^−1^ for both patients). Blue = patient 1, red = patient 2; green = difference between patient 1 and 2; dark grey = inspiratory branch of the ventilator, light grey = expiratory branch of the ventilator. (**A**) Flows at Y-connectors. (**B**) Flows at inspiratory branch of patients. (**C**) Flows at expiratory branch of patients. (**D**) Flows at inspiratory and expiratory branches of ventilator. (**E**) Volumes at Y-connectors. (**F**) Volumes at inspiratory branch of patients. (**G**) Volumes at expiratory branch of patients. (**H**) Volumes at inspiratory and expiratory branches of ventilator. (**I**) Alveolar pressures. (**J**) Airways pressures at HME.

In the first setting, compliance and resistance were the same for both simulated patients (C = 50 mL × cmH_2_O^−1^ and R = 5 cmH_2_O × L^−1^ × s^−1^) and as shown in Figure 2A–J only minimal differences of volumes, flows and pressures between simulated patients can be noted. The total V_T_ delivered by the ventilator was 925 ± 3 mL, patient 1 received 441 ± 2 mL, while patient 2 received a slightly higher volume (and 455 ± 2 mL, *p* < 0.001) (Figure 2E). The tracings of the flows across the Y-connectors (Figure 2A) and alveolar pressures almost perfectly overlapped (Figure 2I). Small differences between the simulated patients have been noted looking at flows across the other branches of the circuit.

### 3.3. Setting C 50-50/R 5-20

**Figure 3:**
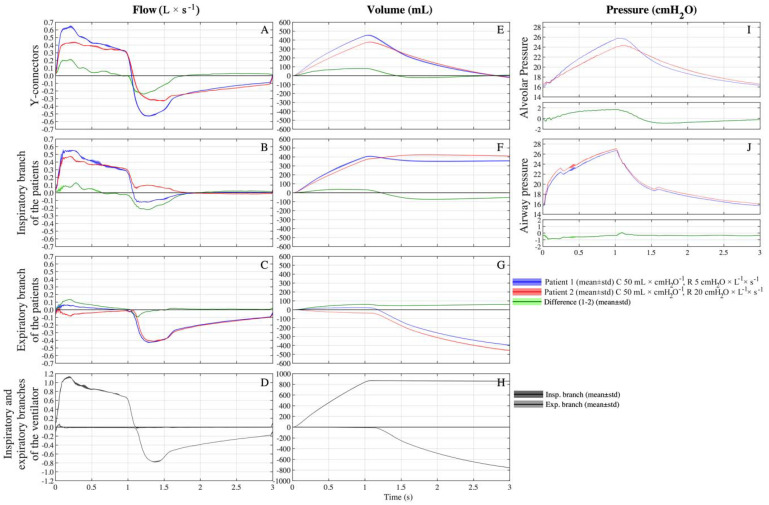
Average (continuous line) ± standard deviation (filled area plot) of multiple breaths of flows, pressures and volumes across the custom-made circuit on setting C 50-50/R 5-20 (C = 50 mL × cmH_2_O^−1^ for both patients, R = 5 cmH_2_O × L^−1^ × s^−1^ for patient 1 and R = 20 cmH_2_O × L^−1^ × s^−1^ for patient 2). Blue = patient 1, red = patient 2; green = difference between patient 1 and 2; dark grey = inspiratory branch of the ventilator, light grey = expiratory branch of the ventilator. (**A**) Flows at Y-connectors. (**B**) Flows at inspiratory branch of patients. (**C**) Flows at expiratory branch of patients. (**D**) Flows at inspiratory and expiratory branches of ventilator. (**E**) Volumes at Y-connectors. (**F**) Volumes at inspiratory branch of patients. (**G**) Volumes at expiratory branch of patients. (**H**) Volumes at inspiratory and expiratory branches of ventilator. (**I**) Alveolar pressures. (**J**) Airways pressures at HME.

**Figure 4:**
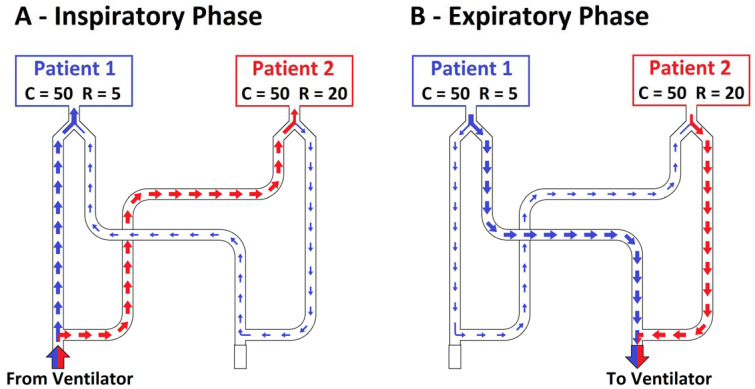
Flows across the custom-made circuit on setting C 50-50/R 5-20 during the inspiratory time (**A**) and the expiratory time (**B**). Blue = patient 1, red = patient 2. Circuit and arrows in figure are not to scale.

In the second setting, compliances were kept fixed to 50 mL × cmH_2_O^−1^ for both simulated patients, and resistance was equal to 5 cmH_2_O × L^−1^ × s^−1^ for patient 1 and 20 cmH_2_O × L^−1^ × s^−1^ for patient 2. The total inspiratory volume measured at the inspiratory branch of the ventilator was equal to 864 ± 5 mL. At the Y-connectors, patient 1 (C50/R5) received a higher V_T_ than patient 2 (455 ± 5 vs. 377 ± 3 mL, *p* < 0.001), approximately 55% of the total volume (Figure 3E). Patient 1 (C50/R5) received approximately 95% of its V_T_ from the inspiratory branch (Figure 3B) while the remaining volume bypassed patient 2 and arrived through the expiratory branches (Figure 3C and Figure 4A).

During the expiratory phase, this phenomenon was reversed, so the expiratory flow of patient 1 was primarily exhaled directly through the patient 1 expiratory branch (Figure 3C) and, to a lesser extent, corresponding to 11% of its V_T_, through patient 1 inspiratory branch, patient 2 inspiratory branch, and, eventually, patient 2 expiratory branch (Figure 4B).

The alveolar pressure of patient 1 was higher during most inspiration but lower during the majority of expiration (Figure 3I). In fact, patient 1 had a lower time constant than patient 2 (0.25 s vs. 1 s), so at the same inspiratory time, patient 2 received a lower percentage of the total inspirable V_T_. Interestingly, at the end of the expiration, the alveolar pressures of both patients were extremely similar (16.32 ± 0.03 and 16.57 ± 0.04 cmH_2_O, patient 1 and 2, respectively).

### 3.4. Setting C 50-30/R 20-5

**Figure 5:**
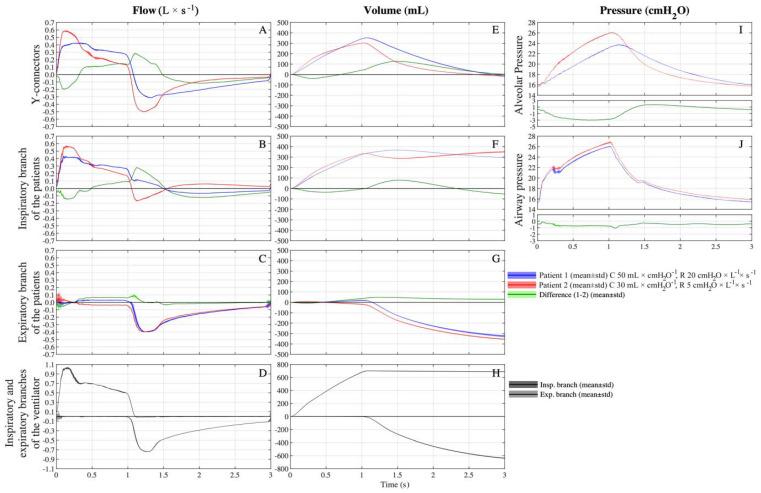
Average (continuous line) ± standard deviation (filled area plot) of multiple breaths of flows, pressures and volumes across the custom-made circuit on setting C 50-30/R 20-5 (C = 50 mL × cmH_2_O^−1^ for patient 1, C = 30 mL/cmH_2_O for patient 2 and R = 20 cmH_2_O × L^−1^ × s^−1^ for both patients). Blue = patient 1, red = patient 2; green = difference between patient 1 and 2; dark grey = inspiratory branch of the ventilator; light grey = expiratory branch of the ventilator. (**A**) Flows at Y-connectors. (**B**) Flows at inspiratory branch of patients. (**C**) Flows at expiratory branch of patients. (**D**) Flows at inspiratory and expiratory branches of ventilator. (**E**) Volumes at Y-connectors. (**F**) Volumes at inspiratory branch of patients. (**G**) Volumes at expiratory branch of patients. (**H**) Volumes at inspiratory and expiratory branches of ventilator. (**I**) Alveolar pressures. (**J**) Airways pressures at HME.

**Figure 6:**
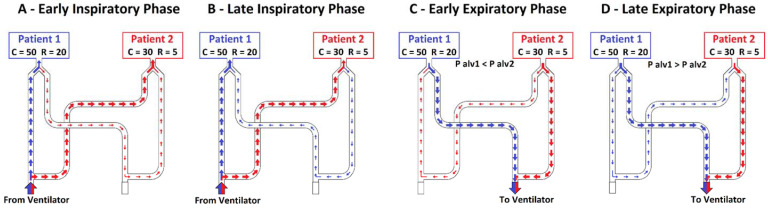
Flows across the custom-made circuit in setting C 50-30/R 20-5 during the inspiratory time (**A**,**B**) and the expiratory time (**C**,**D**). Blue = patient 1, red = patient 2. Circuit and arrows in figure are not to scale.

On the third setting, the compliance was equal to 50 mL × cmH_2_O^−1^ for patient 1 and 30 mL × cmH_2_O^−1^ for patient 2, while the resistance was equal to 20 cmH_2_O × L^−1^ × s^−1^ for patient 1 and to 5 cmH_2_O × L^−1^ × s^−1^ for patient 2. The total volume delivered by the ventilator was 701 ± 4 mL. Patient 1 (C50/R20) received a higher V_T_ (352 ± 3 mL, approximately 50% of the V_T_ delivered by the ventilator) than patient 2 (C30/R20, 303 ± 2 mL) (Figure 5E). Patient 1 had lower absolute inspiratory and expiratory peak flow values compared to patient 2 (0.422 ± 0.006 vs. 0.586 ± 0.006 L/s, *p* < 0.001, Figure 5A). During the first ≈0.3 s of inspiration, patient 2 (C30/R5) received the V_T_ (126/303 mL) from its inspiratory branch (120/126 mL, 95%) and the circuit of patient 1 (6/126 mL, 5%). For the remaining part of the inspiration, patient 1 (C50/R20) received the further V_T_ (263/352 mL) from its inspiratory branch (245/263 mL, 93%) and the circuit of patient 2 (18/263 mL, 7%) (Figure 5C and Figure 6A,B). The net V_T_ received by patient 1 was 96% (337 mL) from the inspiratory branch while the remaining volume arrived through the expiratory branches.

The alveolar pressure of patient 2, compared to patient 1, was higher during inspiration and lower during expiration (Figure 5I). During the first part of the expiration, patient 2 (C30/R5) expired through its expiratory branch and the circuit of patient 1 (30.4 mL); subsequently, patient 1 (C50/R20) expired through its expiratory branch and the circuit of patient 2 (70.9 mL) (Figure 5B and Figure 6C,D). The net volume expired by patient 1 was 96% (340 ± 4 mL) from its expiratory branch while the remaining volume passes through the inspiratory branches of the patients (40.5 ± 0.4 mL).

### 3.5. Settings C 50-30/R 20-20 and C 50-30/R 5-20

See Additional Results and Figures S1–S4 in the Supplementary Material for further details.

## 4. Discussion

In this experimental study we evaluated the flows, pressure and mechanics of two simulated patients simultaneously connected to the same ventilator with a simple custom-made ventilatory circuit without one-way valves. We confirmed that any difference in compliances and airway resistances between the two patients ventilated with the same ventilator on a pressure-controlled mode could result in a wide variation in minute ventilation and alveolar pressures [14]. Interestingly, we recorded frequent interactions and complex cross-flows between the simulated patients in all the tested combinations of compliances and resistances, during both inspiration and expiration. Up to 20% of the V_T_ delivered to one patient flowed through the other patient’s respiratory circuit.

As expected during pressure-controlled ventilation, a reduction in compliance or an increase in resistance always reduced the V_T_ delivered to the patient. Strikingly, end-expiratory alveolar pressure (minimal) was almost constant and not affected by mechanical characteristics.

During the COVID-19 pandemic, ICU beds and mechanical ventilators have been limited resources, and eventually, extreme solutions as ventilation of two patients with one ventilator have been applied [9]. Although several authors have previously described ventilator sharing, we felt the need to evaluate further the interaction between two patients supported by a single ventilator, focusing on cross-flows between patients.

In 2006, Neyman and colleagues ventilated four test lungs with one ventilator [6]. This study demonstrated the feasibility of simultaneous ventilation, both with pressure- and volume-controlled modes. Nevertheless, the authors did not investigate the pressures, flows, and volumes across the different sectors of the ventilator circuit. Subsequently, Paladino and colleagues ventilated for 12 h, four adult healthy sheep with a single ICU mechanical ventilator [11]. The authors reported significant episodes of hypoxemia and hypercapnia, as well as the contamination of the expiratory branch of the respiratory circuits.

In a further in vitro study, Branson and colleagues assessed the V_T_ delivered by one ventilator to four pulmonary simulators with different respiratory mechanics (compliance and airway resistance) [10]. Equivalent V_Ts_ were provided only when the respiratory characteristics of the simulators were identical, otherwise, discrepancies in the V_T_ were detected during both pressure- and volume-controlled ventilation modes. The authors reported that changes in resistances alone resulted in a variable V_T_ to a lesser extent compared to compliance changes. Variation of both compliance and resistance resulted in wide variations in delivered V_T_.

When the two simulated patients had the same characteristics of respiratory mechanics, the delivered V_T_ during pressure-controlled ventilation was similar, and the cross-flows between patients were limited. When the respiratory mechanics, compliance or resistances, or both, are different, minute ventilation and alveolar pressures may be quite different, eventually resulting in acute deleterious complications for the patients. Moreover, we observed an abnormal distribution of flows within the circuit with the appearance of complex cross-flows between patients. Indeed, during inspiration, one patient could receive the V_T_ from his/her inspiratory branch of the circuit and, to a lesser extent, from his/her expiratory branch. In this latter case, such volume also travelled through the inspiratory and expiratory branches of the other patient. Similarly, during expiration, the expired volume could reach the ventilator through both the inspiratory and expiratory branches. Multiple inversions of these cross-flows were recorded within the same breath when both compliance and resistance of the two patients were different. Cross-flow interactions may expose patients to complications like CO_2_ rebreathing due to increased apparatus dead space.

We found, similarly to several investigators, that patients characterized by a higher compliance of respiratory system always received a higher V_T_ than patients with a lower compliance [4,10,14,17,24]. The patient with lower resistance also received, although to a smaller extent, a higher V_T_ than the patient with higher resistance.

The interaction between the two patients and one ventilator is complex, difficult to predict and potentially harmful. Patients with different respiratory system characteristics may receive inadequate ventilation, and consequently, the gas exchange might be significantly compromised. Indeed, during the shared ventilation of four healthy sheep, Paladino and colleagues observed marked hypoxemia and hypercarbia [11]. However, Beitler and colleagues successfully applied shared ventilation for two days to selected patients with compatible characteristics [9].

Recently, many investigators have tried to optimize sharing ventilation by modifying the ventilatory circuits with less or more complicated approaches to ensure greater individualized ventilation and increase the safety profile [14,16,25,26,27,28,29,30,31,32,33,34,35,36]. These strategies have been analyzed in silico, in vitro or in vivo to assess the potential safety and feasibility of ventilatory sharing [15,37,38].

Many of these techniques applied two or more one-way valves, flow resistors, or pressure limiting valves to prevent cross- or backflows and allow the delivered V_T_ to each patient to be individualized [39]. Encouraging results have been reported, although the increased complexity of the proposed solution may reduce prompt applications and expose the patients to extremely dangerous complications in the case of malfunction or misplacement. Furthermore, we cannot exclude that the absence of one-way valves in our setup, allowing free flows between patients, limited the diversity of the alveolar pressures between the two patients despite very different time constants. In fact, at the end of the expiration, the measured alveolar pressures of both patients were quite similar to the set PEEP level.

It is our opinion that sharing a mechanical ventilator should be limited to extreme necessity and dictated by desperate conditions. This strategy should be used in selected patients only for a very short period, under close clinical surveillance by trained personnel until a new ventilator does become available. The monitoring of the delivered V_T_ and end-tidal CO_2_ at the Y-connector of each patient is strongly recommended. Bacterial/viral filters on both patient circuits are essential to reduce the risk of cross-contamination.

However, we believe that studying and optimizing ventilator sharing is relevant, also considering that during the pandemic, some of the purchased medical instruments, including mechanical ventilators, were sometimes proved not capable of properly supporting severe respiratory failure in COVID-19 patients [40].

Our study has numerous limitations that deserve to be mentioned. First, this is an experimental in vitro study performed on passive lung simulators. Second, the study was conducted during the COVID-19 outbreak when resources were minimal, thus only the employed anesthesia machine was available for the test. Third, only one device and one ventilator setting were evaluated. We tested only pressure-controlled ventilation, which is, in our opinion, the safest mode for shared ventilation. Fourth, in our data acquisition system there was only one pneumotachograph, thus flow measurements in the different circuit sections were not simultaneous, promoting possible measurement errors. Fifth, we evaluated a limited number of compliances and airway resistances combinations. Moreover, we did not test setups with different inspiratory and expiratory resistances, active humidification, or malfunction of heat and moisture exchangers.

## 5. Conclusions

The simultaneous ventilation of two or more patients with one ventilator is a complex procedure that could result in wide discrepancies of minute ventilation and alveolar pressures between patients. The use of a simple ventilator circuit without one-way valves exposes patients to constant interactions and intricate cross-flows.

Planning the emergency responses to health crises should always be the prime strategy so that the “sharing ventilation” option should never be required.

## Figures and Tables

**Table 1:** Mechanical characteristics of the 2 “patients” in the 5 different settings tested in the study. C = compliance (mL × cmH_2_O^−1^); R = resistance (cmH_2_O × L^−1^ × s^−1^); τ = time constant (s).

Setting	C 50-50/R 5-5			C 50-50/R 5-20			C 50-30/R 20-20			C 50-30/R 5-20			C 50-30/R 20-5		
	C	R	τ	C	R	τ	C	R	τ	C	R	τ	C	R	τ
Patient 1	50	5	0.25	50	5	0.25	50	20	1.00	50	5	0.25	50	20	1.00
Patient 2	50	5	0.25	50	20	1.00	30	20	0.60	30	20	0.60	30	5	0.15

**Table 2:** Mean + standard deviation values of tidal volumes measured at Y-connectors of the patients, minimum and maximum values of alveolar pressures and cross-volumes between the 2 patients measured during the inspiratory and expiratory times in the 5 different settings tested in the study. *p* refers to *t*-test to compare the average values between the 2 patients. C = compliance, R = resistance, Pt = patient. E1 indicates early expiratory phase from 1.08 to 1.37 s; L1 indicates late expiratory phase from 1.37 to 3.00 s; E2 indicates early inspiratory phase from 0.00 to 0.26 s; L2 indicates late inspiratory phase from 0.26 to 1.07 s; E3 indicates early expiratory from 1.07 to 1.49 s; L3 indicates late expiratory phase from 1.49 to 3.00 s. Grey cells indicates negligible cross-volume values.

Setting	Pt	Tidal Volume at Y-Connectors (mL)	*p*	Min. Alveolar Pressure (cmH_2_O)	*p*	Max. Alveolar Pressure (cmH_2_O)	*p*	Inspiratory Time Cross-Volumes (mL)		Expiratory Time Cross-Volumes (mL)	
**C 50-50/** **R 5-5**	**1**	441 ± 2	<0.001	16.26 ± 0.01	0.286	25.33 ± 0.01	0.673				
	**2**	455 ± 2		16.24 ± 0.03		25.32 ± 0.05					
**C 50-50/** **R 5-20**	**1**	455 ± 5	<0.001	16.32 ± 0.03	<0.001	25.78 ± 0.02	<0.001	23.8 mL from Pt 2 to Pt 1		50.4 mL from Pt 1 to Pt 2	
	**2**	377 ± 3		16.57 ± 0.04		24.33 ± 0.03					
**C 50-30/** **R 20-20**	**1**	372 ± 2	<0.001	15.88 ± 0.03	<0.001	23.52 ± 0.05	<0.001	26.6 mL from Pt 2 to Pt 1		7.5 mL from Pt 2 to Pt 1 ^E1^	54.9 mL from Pt 1 to Pt 2 ^L1^
	**2**	283 ± 2		15.5 ± 0.04		25.05 ± 0.07					
**C 50-30/** **R 5-20**	**1**	473 ± 4	<0.001	15.73 ± 0.03	<0.001	24.84 ± 0.02	<0.001	33.6 mL from Pt 2 to Pt 1		92.9 mL from Pt 1 to Pt 2	
	**2**	269 ± 3		15.5 ± 0.04		25.05 ± 0.04					
**C 50-30/** **R 20-5**	**1**	352 ± 3	<0.001	15.94 ± 0.02	<0.001	23.75 ± 0.04	<0.001	6.1 mL from Pt 1 to Pt 2 ^E2^	18 mL from Pt 2 to Pt 1 ^L2^	30.4 mL from Pt 2 to Pt 1 ^E3^	70.9 mL from Pt 1 to Pt 2 ^L3^
	**2**	303 ± 2		15.7 ± 0.03		26.05 ± 0.07					

## Data Availability

The dataset used and/or analyzed during the current study are available from the corresponding author on reasonable request.

## Supplementary Materials

The following are available online at https://www.mdpi.com/article/10.3390/membranes11070547/s1, The Online Supplement: membranes-11-00547-supplementary_R2.doc. Figure S1: average breath tracings of setting C 50-30/R 20-20, Figure S2: Flows across the circuit in setting C 50-30/R 20-20, Figure S3: average breath tracings of setting C 50-30/R 5-20, Figure S4: Flows across the circuit in setting C 50-30/R 5-20.

## Abbreviations

C	compliance
CO_2_	carbon dioxide
HME	heat and moisture exchanger
ICU	intensive care unit
PCV	pressure-controlled ventilation
P alv	alveolar pressure
P aw	airway pressure
PEEP	positive end expiratory pressure
Pt	patient
R	resistance
RR	respiratory rate
τ	time constant
V_T_	tidal volume
