# Supplementary material for: Sharing Mechanical Ventilator: In Vitro Evaluation of Circuit Cross-Flows and Patient Interactions
Source: Membranes (Basel). 2021 Jul 20;11(7):547. doi: 10.3390/membranes11070547 (PMC8307053; doi:10.3390/membranes11070547)
Supplement: Supplementary file 1 [file membranes-11-00547-s001.zip › membranes-1269785-supplementary.pdf]

Supplementary information

Sharing Mechanical Ventilator: In Vitro Evaluation of Circuit Cross-Flows and Patient Interactions

Sebastiano Maria Colombo [1,2,†], Michele Battistin [3,†], Eleonora Carlesso [2], Luigi Vivona [2], Fabio Carfagna [4], Carlo Valsecchi [1], Gaetano Florio [2], Luca Carenzo [4], Tommaso Tonetti [5], Vito Marco Ranieri [5], Maurizio Cecconi [4], Antonio Pesenti [1,2], Giacomo Grasselli [1,2] and Alberto Zanella [1,2,*]

[1] Department of Anaesthesia and Intensive Care Medicine, Fondazione IRCCS Ca' Granda Ospedale Maggiore Policlinico, 20122, Milan, Italy; sebastiano.colombo@gmail.com (S.M.C.); carlovalsecchi5@gmail.com (C.V.); antonio.pesenti@unimi.it (A.P.); giacomo.grasselli@unimi.it (G.G.)
[2] Department of Pathophysiology and Transplantation, University of Milan, 20122, Milan, Italy; eleonora.carlesso@unimi.it (E.C.); luigi.vivona@unimi.it (L.V.); g.floriomed@gmail.com (G.F.)
[3] Fondazione IRCCS Ca' Granda Ospedale Maggiore Policlinico, Center for Preclinical Research, 20122, Milan, Italy; battistin.michele@gmail.com
[4] Department of Anaesthesia and Intensive Care Medicine Rozzano, IRCCS Humanitas Research Hospital, 20089, Rozzano (Milano), Italy; fabio.carfagna@hunimed.eu (F.C.); luca.carenzo@gmail.com (L.C.); maurizio.cecconi@hunimed.eu (M.C.)
[5] Dipartimento di Scienze Mediche e Chirurgiche Bologna, Alma Mater Studiorum University of Bologna, 40126, Bologna, Italy; tommaso.tonetti@unibo.it (T.T.); m.ranieri@unibo.it (V.M.R.)
* Correspondence: alberto.zanella1@unimi.it; Tel.: +0255033285
† These authors contributed equally.

Setting C 50-30 / R 20-20

In the third setting, resistances were kept constant at 20 $cmH_2O \times L^{-1} \times sec^{-1}$ for both simulated patients while compliance was equal to 50 $mL \times cmH_2O^{-1}$ for patient 1 and 30 $mL \times cmH_2O^{-1}$ for patient 2. The total inspiratory volume delivered by the ventilator was 664 ± 3 mL. Patient 1 received an higher tidal volume (372 2 mL, nearly 56% of the total volume) than patient 2 (283 2 mL, $p<0.001$) (Supplementary Material Figure S1E). Patient 1 (C 50 / R 20) received approximately 93% of its tidal volume from the inspiratory branch (Supplementary Material Figure S1F) while the remaining volume bypassed patient 2 and arrived through the expiratory branches (Supplementary Material Figure S1G and Figure S2A). This cross-flow was almost continuous over the entire inspiration (Supplementary Material Figure S1C).

At the end of the inspiration, the alveolar pressure of patient 2 was higher, mainly due to its lower time constant (Supplementary Material Figure S1I).

This difference in alveolar pressures also affected the expiratory flow kinetics (Supplementary Material Figure S2B-C for major details). During the first part of the expiration, patient 2 (C 30 / R 20) expired mainly through its expiratory branches but also through its inspiratory branch and patient 1 branches (approximately 7.5 mL, 2% of patient 1 tidal volume). During the second part of the expiration, patient 1 (C 50 / R 20) expired both through its expiratory branches and its inspiratory branch and patient 2 branches (54.9 mL, 15% of patient 1 tidal volume) (Supplementary Material Figure S1A-B, J). The net amount is approximately equal to 47 mL (13%) through patient 2 branches.

Figure S1

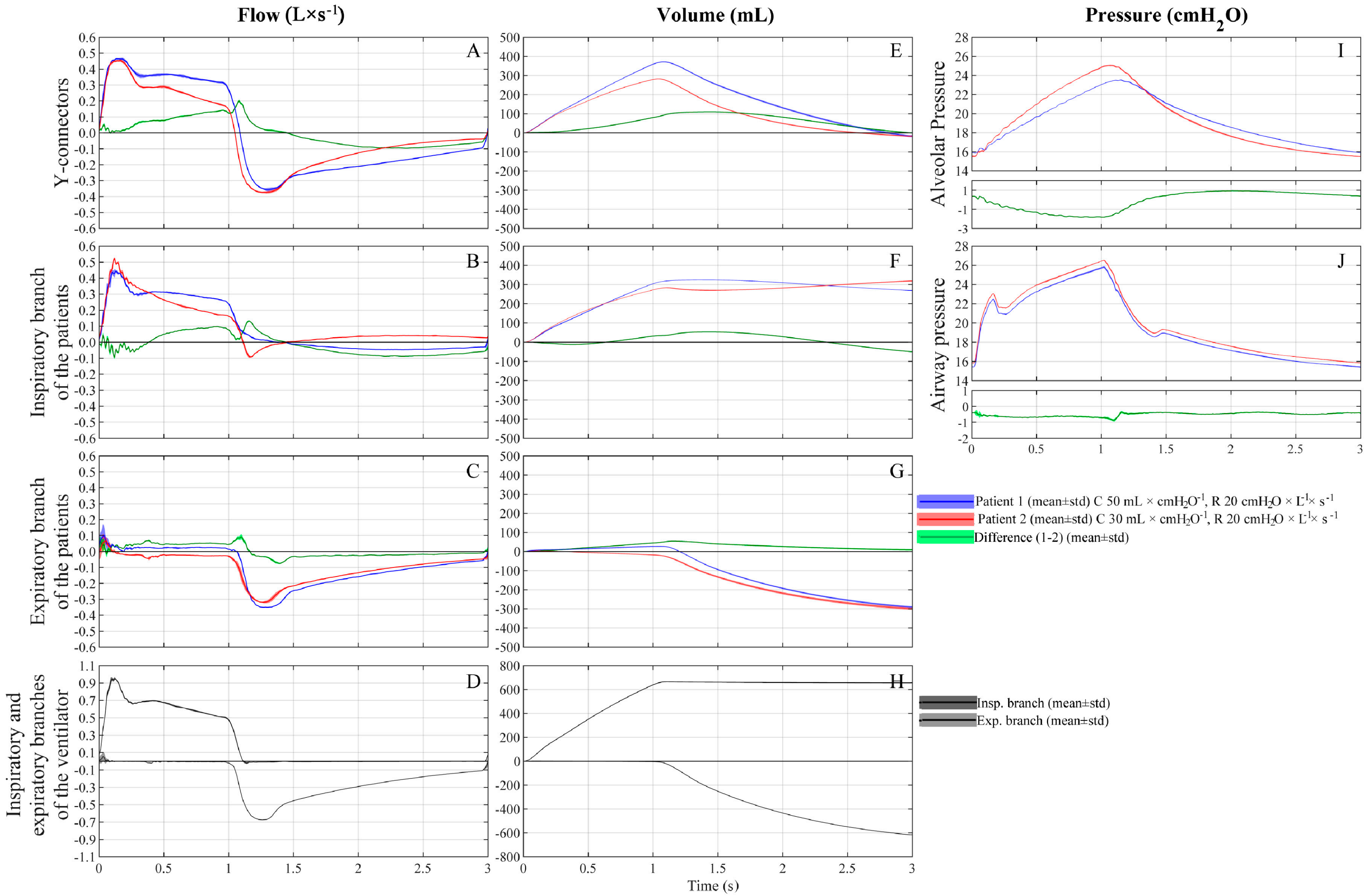

Flow (L×s⁻¹)
Volume (mL)
Pressure (cmH2O)
Y-connectors
Inspiratory branch of the patients
Expiratory branch of the patients
Inspiratory and expiratory branches of the ventilator
Alveolar Pressure
Airway pressure
A
B
C
D
E
F
G
H
I
J
Time (s)
Patient 1 (mean±std) C 50 mL × cmH2O-1, R 20 cmH2O × L-1× s-1
Patient 2 (mean±std) C 30 mL × cmH2O-1, R 20 cmH2O × L-1× s-1
Difference (1-2) (mean±std)
Insp. branch (mean±std)
Exp. branch (mean±std)

Figure S1: average breath tracings of setting C 50-30 / R 20-20

Average (continuous line) ± standard deviation (filled area plot) of multiple breaths of flows, pressures and volumes across the custom-made circuit in setting C 50-30 / R 20-20 (C = 50 $mL \times cmH_2O^{-1}$ for patient 1, C = 30 $mL \times cmH_2O^{-1}$ for patient 2 and R = 20 $cmH_2O \times L^{-1} \times sec^{-1}$ for both patients). Blue = patient 1, Red = patient 2; Green = difference between patient 1 and 2; Dark grey = inspiratory branch of the ventilator, Light grey = expiratory branch of the ventilator. A - Flows at Y-connectors. B – Flows at inspiratory branch of patients. C - Flows at expiratory branch of patients. D - Flows at inspiratory and expiratory branches of ventilator. E - Volumes at Y-connectors. F – Volumes at inspiratory branch of patients. G - Volumes at expiratory branch of patients. H – Volumes at inspiratory and expiratory branches of ventilator. I – Alveolar pressures. J – Airways pressures at HME.

Figure S2

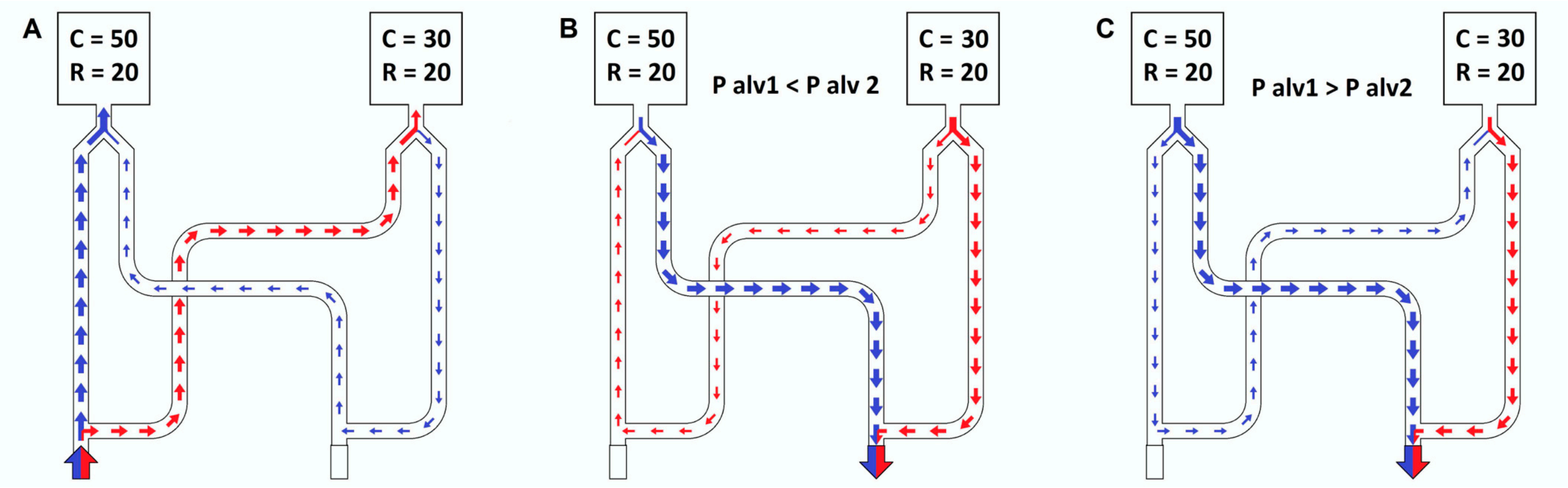

Figure S2: Flows across the circuit in setting C 50-30 / R 20-20

Flows across the custom-made circuit in setting C 50-30 / R 20-20 during the inspiratory time (A) and the expiratory time (B and C). Blue = patient 1, Red = patient 2. Circuit and arrows in figure are not in scale.

Setting C 50-30 / R 5-20

In the fourth setting, compliance was equal to 50 mL×cmH_2O^{-1} for patient 1 and 30 mL×cmH_2O^{-1} for patient 2, while resistance was equal to 5 cmH_2O×L^{-1}×sec^{-1} for patient 1 and to 20 cmH_2O×L^{-1}×sec^{-1} for patient 2. The total volume delivered by the ventilator was 740 ± 3 mL. Patient 1 (C 50 / R 5) received an higher tidal volume (473 4 mL, nearly 64% of the total tidal volume) than patient 2 (C 30 / R 20) (269 3 mL, $p<0.001$) (Supplementary Material Figure S3E). Patient 1 received approximately 85% of its tidal volume from the inspiratory branch (Supplementary Material Figure S3B) while the remaining volume arrived through the expiratory branches (Supplementary Material Figure S3C, and Figure S4A). Similarly, the majority (approximately 80%) of expired volume from patient 1 reached the ventilator through its own expiratory branch, while the remaining 20% through its inspiratory branch and the circuit of patient 2 (Supplementary Material Figure S4B for major details). The alveolar pressures of the patients were almost identical (Supplementary Material Figure S3I).

Figure S3

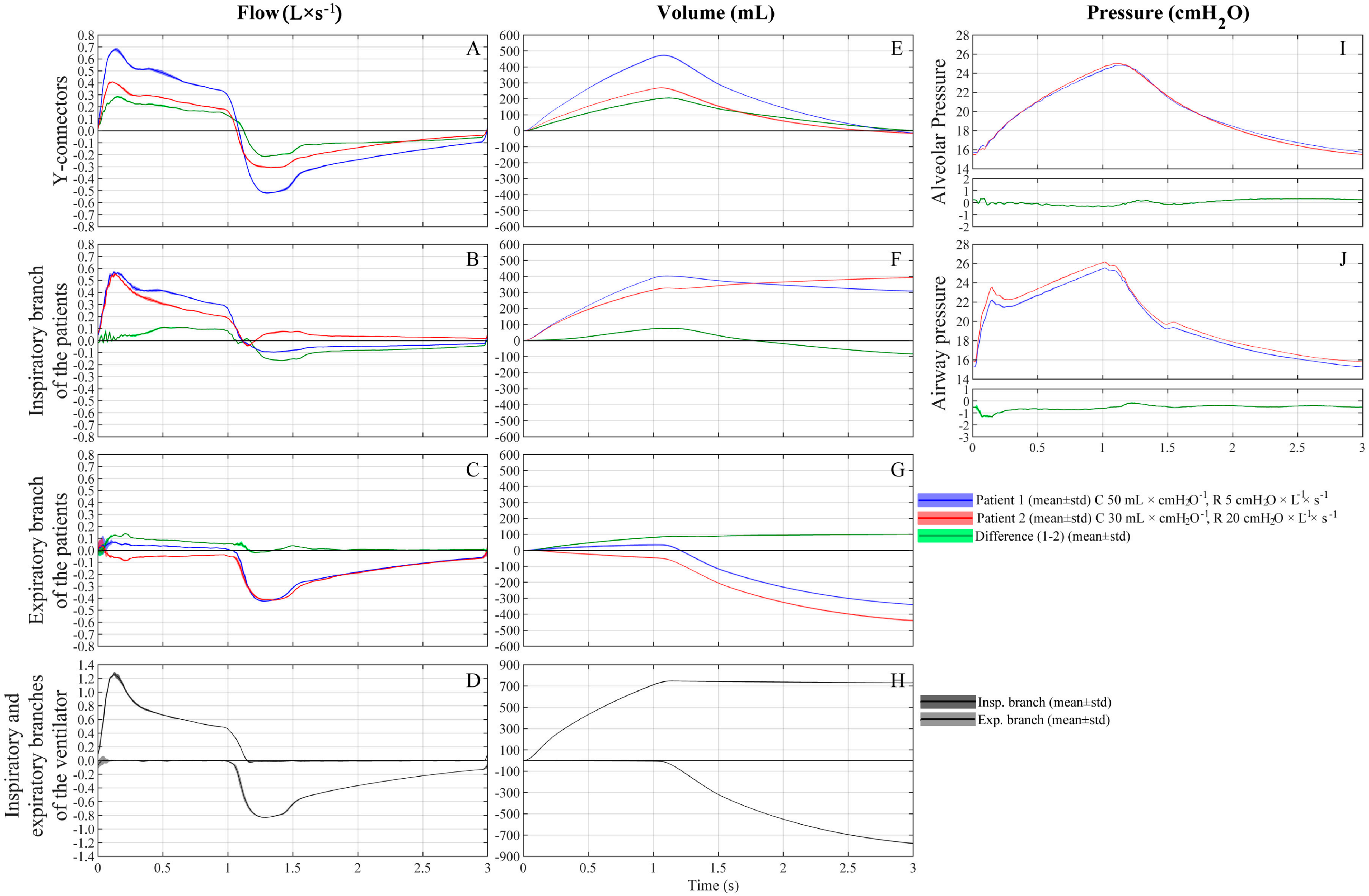
Flow (L×s-1)
Volume (mL)
Pressure (cmH2O)
Y-connectors
Inspiratory branch of the patients
Expiratory branch of the patients
Inspiratory and expiratory branches of the ventilator
Alveolar Pressure
Airway pressure
A
B
C
D
E
F
G
H
I
J
Time (s)
Patient 1 (mean±std) C 50 mL × cmH2O-1, R 5 cmH2O × L-1× s-1
Patient 2 (mean±std) C 30 mL × cmH2O-1, R 20 cmH2O × L-1× s-1
Difference (1-2) (mean±std)
Insp. branch (mean±std)
Exp. branch (mean±std)

Figure S3: average breath tracings of setting C 50-30 / R 5-20

Average (continuous line) ± standard deviation (filled area plot) of multiple breaths of flows, pressures and volumes across the custom-made circuit in setting C 50-30 / R 5-20 (C = 50 mL×cmH_2O^{-1} for patient 1, C = 30 mL×cmH_2O^{-1} for patient 2 and R=5 cmH_2O×L^{-1}×sec^{-1} for patient 1 and to 20 cmH_2O×L^{-1}×sec^{-1} for patient 2). Blue represents patient 1; Red represents patient 2; Green represents difference between patient 1 and 2; Dark grey represents the inspiratory branch of the ventilator; Light grey represents the expiratory branch of the ventilator. A - Flows at Y-connectors. B – Flows at inspiratory branch of patients. C - Flows at expiratory branch of patients. D - Flows at inspiratory and expiratory branches of ventilator. E - Volumes at Y-connectors. F – Volumes at inspiratory branch of patients. G - Volumes at expiratory branch of patients. H – Volumes at inspiratory and expiratory branches of ventilator. I – Alveolar pressures. J – Airways pressures at HME.

Figure S4

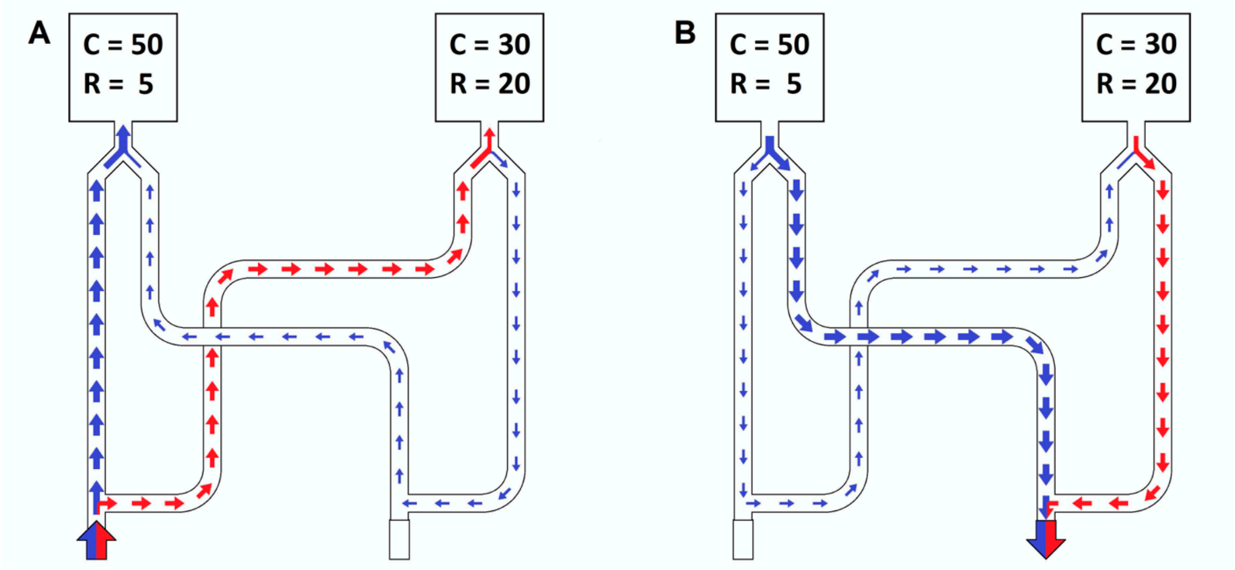

Figure S4: Flows across the circuit in setting C 50-30 / R 5-20

Flows across the custom-made circuit in setting C 50-30 / R 5-20 during the inspiratory time (A) and the expiratory time (B). Blue = patient 1, Red = patient 2. Circuit and arrows in figure are not in scale.
